# Factors influencing the attitudes of young Sri Lankan-Australians towards seeking mental healthcare: a national online survey

**DOI:** 10.1186/s12889-022-12842-5

**Published:** 2022-03-19

**Authors:** Chethana Mudunna, Josefine Antoniades, Thach Tran, Jane Fisher

**Affiliations:** 1 Monash University, 553 St. Kilda Road, Melbourne, VIC 3004 Australia; 2 National Ageing and Research Institute, 34-54 Poplar Road, Parkville, VIC 3052 Australia

**Keywords:** Sri Lankan-Australians, Attitudes about mental health problems, Stigma, Mental healthcare

## Abstract

**Background:**

Sri Lankans, as part of the South Asian diaspora, comprise one of the largest migrant groups in Australia. Although few data are available, South Asian migrants appear to experience higher rates of mental health problems, but seek help at lower rates than other migrant groups. Understanding factors that underpin mental health care seeking is necessary to inform mental health promotion strategies, including access to care. The aim was to investigate factors influencing attitudes to seeking care for mental health problems among young Sri Lankan-Australians born in Sri Lanka or in Australia.

**Methods:**

An anonymous cross- sectional online survey which included the Multiethnic Identity Measure, Perceived Stigma Questionnaire, General Help-Seeking Questionnaire, Attitudes Towards Seeking Professional Psychological Help questionnaire and study specific questions to ascertain sociodemographic characteristics. The survey was advertised on social media and specifically included young adults aged between 18–30, who self-identified as being of Sri Lankan heritage and were living in Australia. Participation was not possible for those that did not have access to the internet. Data were analyzed using bivariable and multivariable statistics.

**Results:**

Of the 396 people who attempted the survey, 323 provided fully completed data, 2 provided > 50% completed data which were included in the analyses. 71 were excluded because < 50% of the survey was completed. From all participants, 39.70% were born in Australia (SLaus), 54.46% born in Sri Lanka (SLsl) and 5.8% born in other countries. SLsl had more stigmatizing attitudes towards mental illness (*p* = 0.027) and seeking professional psychological help (*p* = 0.03). Women, those who had spent more years living in Australia and whose fathers were more highly educated had less stigmatized attitudes toward mental illness and help-seeking.

**Conclusion:**

Country of birth, family characteristics and gender influence stigma towards mental illness and help-seeking. Public health strategies to promote understanding of mental health problems and seeking mental healthcare are more likely to be effective if they address these factors directly.

## Background

Since 2000 the number of international migrants has increased from 174 million people in 2000 to 272 million people in 2019 with almost 3.5% of the world’s population living in a country other than their country of birth [1]. The migratory process in which people travel from one country, region or place of residence to settle in another, can be temporary, permanent, recurrent or seasonal [2, 3]. The overall process may concern individuals or a group of people and can be driven by socioeconomic, educational or political factors [3]. Therefore, migrants experience diverse experiences throughout the migratory process.

Migrants may be particularly vulnerable to experiencing mental health problems (MHPs) because of the adaptive challenges of acquiring and using another language, economic hardship and learning to live in another culture and navigate an unfamiliar health system [4–6]. This is of particular concern in the Australian context as more than a quarter of the Australian population (29.8%) are born overseas [7].

Migrants from South Asia appear to experience higher rates of MHPs than migrants from other regions of the world [4, 5, 8–10]. While the underpinning causes remains unclear, research shows that South Asian migrants in high-income countries have low help-seeking rates for MHPs relative to the general community [5, 8, 9, 11]. Further, a significant barrier to understanding the prevalence of MHPs in Sri Lankan migrants may be attributed to how data is collected and reported where frequently Sri Lankan migrants and other Asian subpopulations are aggregated under the umbrella term ‘Asian’ or in some cases ‘South Asian’ [5]. This data aggregation obscures potential differences in prevalence, access to services, attitudes and health behaviours between ethnic groups [12–16]. In the Australian context, research suggest that there are significant differences in the degree of psychological distress among Culturally and Linguistically Diverse (CALD) groups, which allude to underpinning vulnerabilities in certain populations. However, due to data aggregation it is not possible to identify specific populations [17].

In 2018–2019, 29.2% of all migrants to Australia were South Asian [18] and Sri Lankan migrants represent the 13^th^ largest overseas- born group in Australia and the second largest South Asian migrant group [19].

Among South Asian migrants, one of the most significant barriers to help-seeking appears to be culturally derived illness beliefs [5, 8, 9]. Such illness beliefs not only affect perceptions towards MHPs and help-seeking trajectories, they can also shape how symptoms are expressed. For example, in some Asian and South Asian cultures psychological symptoms may be expressed through somatisation, a process where psychological distress is expressed as non-specific physical symptoms [20–22]. This can lead to diagnostic inaccuracy by clinicians, which consequently delays access to appropriate treatment, like seeking medical help instead of psychological help for psychological illnesses [23]. Further to this, culture can drive positive or negative reactions towards MHPs which subsequently influence how an individual manages their symptoms or seeks care. In this way culturally derived illness beliefs can influence an individual’s overall illness experience [21]. Therefore, it is important to consider South Asian subcultures as distinct from the broad South Asian population and research the attitudes and help-seeking behaviours in these diverse subcultures in order to develop and implement relevant interventions to improve timely access to mental health services [12–16].

In Asian and South Asian cultures, mental illness stigma may include devaluing, disgracing and disfavouring individuals who have symptoms of a mental illness or have been labelled as mentally ill [24]. The two main categories of stigma includes public stigma, which refers to a society’s discriminatory response towards people with mental illness and self-stigma which can be the internalization of public stigma [13, 24, 25]. The decision to seek help is the end result of a process heavily influenced by these beliefs and attitudes towards mental illnesses and their treatment [13]. Presence of mental illness stigma can lead to delayed help-seeking and diagnosis which have negative implications for timely treatment for MHPs [12–14].

Most of the current evidence about the mental health of Sri Lankan young adults is based on research conducted in Sri Lanka. Although these data indicate that Sri Lankan young people experience higher rates of psychological problems in comparison to their other South Asian counterparts, national data collection has been compromised by the 2004 tsunami and civil war regional ascertainment from Sri Lankans in rural areas by local conflicts. While national estimates have found relatively low rates of MHPs overall, incidence of psychological distress among young Sri Lankans is significantly higher [26].

There is relatively little research about Sri Lankan young people in Australia. However, Antoniades [6, 27] found that mental illness stigma is evident among Sri Lankan-Australians. Yet specific research to better understand the attitudes and beliefs about MHPs among young Sri Lankans living in Australia is needed to better tailor programs and services to young people from culturally diverse backgrounds. Therefore, the aim of this study was to compare the attitudes and beliefs about MHPs and mental healthcare seeking between Sri Lankan young people born in Sri Lanka and those born in Australia.

## Methods

### Design

An online survey was conducted from August 2020 to September 2020 via Qualtrics, targeting Sri Lankan young adults living in Australia.

### Participants

To be included participants had to be aged between 18–30 years, live in Australia and self-identifying as being of Sri Lankan heritage. Participation was not possible for people who were not fluent in written English or did not have access to the internet.

### Recruitment

Participants were recruited via two stages. Initially, a purposive sampling strategy was employed, followed by snowball sampling to allow for a large sample size to be collected.

Specifically, young Sri Lankan- Australians were contacted through four methods. First, 27 Sri Lankan Student Unions at Australian Universities across all states and territories were contacted via e-mail. Respondents were provided with the survey link to post on their social media pages. Secondly, International Student Unions of 37 Australian Universities were contacted in a similar manner and respondents were provided with the survey link. Third, the survey link was posted on 23 public Sri Lankan Facebook groups and for two closed groups, the administrative teams were contacted and provided with the survey link. Last, the research team disseminated information about the study via their professional networks including Sri Lankan and CALD specific organisations and community groups.

### Data sources

Data were collected through a structured anonymous online survey consisting of study specific questions and four widely used standardized short questionnaires assessing ethnic identity, attitude towards mental health problems and help-seeking behavior.

### Stigma towards mental illnesses

Attitudes towards mental health problems was measured using the devaluation-discrimination subscale of the Perceived Stigma Questionnaire (PSQ) [28]. The PSQ is a 29-item questionnaire created to measure perceived stigma of participants on four subscales. The devaluation-discrimination subscale was employed because it has been used cross-culturally to measures the extent to which participants may discriminate against persons living with mental illness [29]. The secrecy, withdrawal and education subscales measure how individuals cope with mental illness. As this study primarily focuses on attitudes towards MHPs, these subscales were not used. Instead, only the devaluation-discrimination subscale was used in this study to measure how participants may discriminate against persons with mental illness. The wording of the original scale was modified in this study to reflect the opinion of the respondent with regards to what they would do as opposed to what most people would do. Furthermore, the original wording of ‘mental hospital’ was modified to ‘psychiatric unit’ as this is a more commonly used term in Australia. The scale has 12 items scored on a six-point Likert Scale ranging from 1 (Strongly Disagree) to 6 (Strongly Agree). Items 5, 6, 7, 9 and 11 are reversed scored therefore a higher mean scale score indicates more positive attitudes towards mental illness. A lower mean scale score indicates a higher level of stigma.

### Attitudes towards seeking mental healthcare

Two standardized measures were used to examine participants’ attitudes towards seeking mental healthcare.

Section 1 of the General Help-Seeking Questionnaire (GHSQ) [30] was used to measure the likelihood of participants seeking help for personal & emotional problems. Section 2 of the GHSQ was excluded as it relates to participants experiencing suicidal ideations. The scale was initially validated among 218 high school students where it was established as a flexible method in measuring help-seeking intentions from a variety of sources that can be applied to a range of contexts [30]. The 10-item questionnaire has a 7-point Likert scale ranging from 1 (Extremely Unlikely) to 7 (Extremely Likely). Items a-d are categorized as informal sources while items e–h are categorized as formal sources. Item ‘I’, ‘I would not seek help from anyone’, was measured separately and was renamed as item ‘no one’. Average scores are calculated for informal and formal sources as well as for item ‘no one’. A score greater than 3.5 indicated a higher likelihood of seeking help from the particular mental healthcare source.

The Attitudes Towards Seeking Professional Psychological Help- Short Form (ATSPPH-SF) [31] was also used to ascertain participants’ general attitudes toward seeking professional psychological help. This instrument is divided into two subscales to examine openness to seek treatment for emotional problems and the perceived value and need in seeking treatment. It is a widely used instrument which is supported in its validity and reliability in a study measuring mental health treatment attitudes of university students and medical patients [32]. This measure has 10-items on a 4-point Likert Scale ranging from 0 (Disagree) to 4 (Agree). A higher subscale score is associated with a more positive attitude towards seeking professional psychological help.

### Ethnic Identity

Ethnic identity was measured using the Multi Ethnic Identity Measure- Revised (‘MEIM-R’) [33]. The MEIM-R [33] is a revised version of the original MEIM [34]. The scale measures the extent to which a person identifies with an ethnic group. It comprises 6-items on a 5-point Likert Scale, 3 of which measure exploration of ethnic identity and the others, commitment to ethnic identity. The scale ranges from 1 (Strongly Disagree) to 5 (Strongly Agree). Higher scores on the exploration subscale suggests greater interest in learning and participating in a given culture and its practices. Higher scores in the commitment scale suggests positive affirmations and great commitment to a particular ethnic group.

In addition, fixed-response option study specific questions were used to assess sociodemographic characteristics, the number of years lived in Australia, language spoken at home and association with Sri Lankan and other ethnic groups in the community. Further, the survey included basic questions: place of birth, parents’ place of birth and parents’ level of education completed.

### Data management and analysis

The primary outcome of the study was stigma towards mental illness and its impact on help-seeking between Sri Lankan young adults born in Sri Lanka and born in Australia.

### Recoding and collapsing

The initial data were recoded and collapsed to form binary variables as follows. Variables were coded as 0 and the italicized reference variables were coded as 1. Gender (other genders, *female*); educational status (no university education, *university education*); relationship status (not single, *single/never married*); household composition (living away from home, *living at home*); unemployed (no, *yes*); employed (no, *yes*); student (no, *yes*); I was born in (other, *Sri Lanka*); my father was born in; (Australia, *Sri Lanka*); my father’s highest level of education completed (no university education, *university education*); my mother was born in (Australia, *Sri Lanka*); my mother’s highest level of education completed (no university education, *university education*); language spoken at home (mostly English, Equally a Sri Lankan Language and English, *mostly a Sri Lankan language*); association in the community (mostly other ethnic groups, *mostly Sri Lankan groups*).

Furthermore, the ATSPPH-SF was recoded following the original instruments coding. 0 = disagree, 1 = partly disagree, 2 = partly agree, 3 = agree.

### Statistical analysis

Statistical analyses were conducted in three stages:

1. Mean scale and subscale scores were calculated for all standardized data tools using the published protocols. Descriptive statistics were calculated for all sociodemographic variables. Chi-squared tests were used to compare significance between participants’ country of birth and categorical demographic variables such as: gender, educational status, relationship status, household composition, employment status, student status, parental country of birth and education, language spoken at home and association in the community. Independent t-tests for continuous variables such as: age, number of years spent in Australia and MEIM-R score, were used to assess significance between these variables and participants’ country of birth.
2. Bivariable analyses were used to examine whether the dependent variable could be explained by the independent variables. In this case, whether country of birth had an effect on sociodemographic characteristics. A bivariate analysis was also conducted on country of birth and mean scale scores of all standardized data tools (PSQ, GHSQ, ATPPHS) to explore whether country of birth influenced attitudes towards MHPs and accessing mental healthcare. ANOVA was used for multiple group mean comparisons. All bivariable analyses included Sri Lankan young adults born in Sri Lanka, born in Australia and those born in other countries who identified as being of Sri Lankan heritage.
3. Multivariable analyses were used to investigate multifactorial determinants and mean scale scores of the PSQ, GHSQ and ATSPPH. For this, the ‘born in other countries’, respondents were excluded from the dataset as the analysis determines the effect of sociodemographic factors on the association between being born in Australia/Sri Lanka and mental illness stigma and help-seeking. The binary forms of the variables were recoded to *Sri Lanka,* Other where ‘*Sri Lanka’* indicates participants born in Sri Lanka and ‘Other’ indicates participants born in Australia. The italicized reference variable was coded as 1.

### Ethics approval

Informed consent was sought from all participants. Participation in the survey was voluntary and participants were informed that consent would be assumed by completing the survey. The study protocol was approved by the Monash University Human Research Ethics Committee (MUHREC 24878).

## Results

### Sociodemographic characteristics

401 people clicked on the survey link. Out of that 396 people attempted the survey. 323 provided fully completed data, 73 provided partial data and 5 clicked on the survey link but did not attempt the survey. For the analyses, 323 fully completed surveys and 2 surveys that were more than 50% completed were included (a total of 325).

The sociodemographic data of Sri Lankan young adults born in Sri Lanka, born in Australia and those born elsewhere are displayed in Table 1.

**Table 1:** Sociodemographic data of Sri Lankan young adults

	**Number (%)**			
	**Sri Lanka**	**Australia**	**Elsewhere**	***p*-value**
**Mean Age in Years (All participants) (± SD)**	23 (± 2.99)			
**Number of years in Australia,** **Mean (All participants) (± SD)**	14.85 (± 8.42)			
**Gender**				**0.005**
Woman	123 (69.49)	85 (65.89)	11 (57.89)	
Man	54 (30.50)	43 (33.33)	7 (36.84)	
Other		1 (0.78)	1 (5.26)	
**Educational Status**				
University Education	155 (87.57)	120 (93.02)	19 (100)	0.114
**Relationship Status**				
Single, Never Married	139 (78.53)	116 (89.92)	18 (94.73)	**0.011**
**Household Composition**				
Living at home, with parents	86 (48.59)	112 (86.82)	16 (84.21)	** < 0.002**
**Unemployed**	64 (36.16)	27 (20.93)	13 (68.42)	**0.015**
**Employed**	110 (62.15)	97 (75.19)	13 (68.42)	0.055
**Student**	125 (70.62)	99 (76.74)	16 (84.21)	> 0.277
**Father Born in Sri Lanka**	175 (98.87)	127 (98.44)	18 (94.73)	**0.037**
**Father’s Educational Status**				
University Education	81 (45.76)	35 (27.13)	12 (63.16)	** < 0.002**
**Mother Born in Sri Lanka**	173 (97.74)	124 (96.12)	16 (84.21)	** < 0.002**
**Mother’s Educational Status**				
University Education	69 (38.98)	39 (30.23)	10 (52.63)	0.529
**Language Spoken at Home**				
Mostly a Sri Lankan Language	95 (53.67)	59 (45.74)	5 (26.32)	0.245
Mostly English	23 (12.99)	21(16.28)	5 (26.32)	
Equally a Sri Lankan Language and English	59 (33.33)	49 (37.98)	9 (47.37)	
**Association within the Community**				
Mostly Sri Lankan Groups	117 (66.10)	82 (63.57)	13 (68.42)	0.860
**MEIM-R, (Mean (± SD))**	3.77 (± 0.72)	3.85 (± 0.77)	3.81 (± 0.46)	0.413

*Note: N* = 325, *MEIM-R* Multi-Ethnic Identity Measure-Revised, Born in Sri Lanka *N* = 177, Born in Australia *N* = 129, Born Elsewhere *N* = 19. Results in bold indicate significant differences between groups. The significance level for gender was measured as females v other genders

### Stigma towards mental illness

The relationship between participants’ country of birth and mean scale scores for the PSQ are shown in Table 2. Sri Lankan young adults born in Sri Lanka (SLsl) had a lower mean scale score, indicating higher stigma compared to Sri Lankan young adults born in Australia (SLaus). Participants born elsewhere showed a lower mean scale score which was more comparable to SLsl. ANOVA test showed that the association between country of birth and perceived stigma towards mental illness was statistically significant.

**Table 2:** Association between country of birth and stigma towards mental illness

	**Sri Lanka**	**Australia**	**Elsewhere**	***p*-value**
**Mean (**± SD) Scale Score	4.76 (± 0.65)	4.95 (± 0.50)	4.73 (± 0.84)	0.027

^*^ANOVA test used to determine *p*-value

### Likelihood of seeking formal and informal mental healthcare

The General Help-Seeking Questionnaire (GHSQ) data were analyzed by participants’ country of birth (see Table 3). Item 9 of the scale- ‘I would not seek help from anyone’, was analyzed separately.

**Table 3:** Association between country of birth and likelihood of seeking help from a mental healthcare source

	**Sri Lanka**	**Australia**	**Elsewhere**	***p*-value**
**Formal Mental Healthcare (Mean (± SD))**	3.82 (± 1.37)	3.48 (± 1.17)	3.45 (± 1.09)	0.058
**Informal Mental Healthcare (Mean (**± **SD))**	4.91 (± 1.07)	4.76 (± 1.24)	4.41 (± 0.76)	0.135
**No One (Mean (**± **SD))**	2.56 (± 1.72)	2.77 (± 1.72)	3.68 (± 1.95)	0.026

^*^ANOVA test used to determine *p*-value

There were no differences in likelihood of seeking informal or formal mental healthcare by country of birth. ANOVA test showed that the association between country of birth and seeking no help was statistically significant.

### Stigma towards professional psychological help-seeking

The results from the Attitudes Towards Seeking Professional Psychological Help (ATSPPH) are shown on Table 4. The value and need in seeking help subscale was significantly associated with participants’ country of birth.

**Table 4:** Association Between Country of Birth and Stigma Towards Professional Psychological Help-Seeking

	**Sri Lanka**	**Australia**	**Elsewhere**	***p*-value**
**Openness to Seek Help (Mean (± SD))**	1.95 (± 0.55)	2.03 (± 0.51)	1.95 (± 0.65)	0.413
**Value and Need in Seeking Help (Mean (**± **SD))**	1.88 (± 0.65)	2.03 (± 0.57)	2.19 (± 0.44)	0.03

^*^ANOVA test used to determine *p*-value

### Sociodemographic characteristics and stigma towards mental illness

A multivariable analysis was used to investigate the association between sociodemographic factors and the Perceived Stigma Questionnaire (PSQ), GHSQ and ATSPPH. Firstly, the stigma towards mental illness was assessed against participants’ sociodemographic characteristics as shown in Table 5. The results suggested that being female was significantly associated with lesser stigmatizing attitudes towards mental illnesses. Similarly, the duration of residence in Australia was associated with lower levels of stigmatizing attitudes (higher mean PSQ).

**Table 5:** The association between stigma towards people with mental illness (PSQ Scale) and sociodemographic characteristics

	**ß**	**95% CI**	***p*-value**
**SLsl**	0.025	-0.185- 0.246	0.781
**Age**	-0.074	-0.045- 0.016	0.342
**Female**	0.179	0.078- 0.375	**0.003**
**University Education**	0.029	-0.169- 0.284	0.620
**Single, Unmarried**	0.005	-0.202- 0.219	0.936
**Living at Home, with Parents**	0.013	-0.187- 0.219	0.875
**Unemployed**	-0.013	-0.455- 0.421	0.939
**Employed**	0.080	-0.328- 0.531	0.643
**Student**	-0.005	-0.191- 0.178	0.943
**Father with University Education**	0.074	-0.063- 0.244	0.246
**Mother with University Education**	0.014	-0.138- 0.172	0.831
**MEIM-R**	0.065	-0.048- 0.152	0.307
**Number of Years in Australia**	0.219	0.001- 0.030	**0.042**
**Language Spoken at Home**	-0.041	-0.104- 0.051	0.503
**Association with Sri Lankan Groups in the Community**	-0.066	-0.233- 0.070	0.291

*N* = 306 (Excluded = 19 respondents from born in other countries category), *MEIM-R* Multi-Ethnic Identity Measure-Revised, Numbers in bold indicate significant results. Refer to methods section to see comparators of variables. Adjusted *r* squared value = 0.058

The comparators for all sociodemographic variables are described in the methods section.

### Sociodemographic characteristics and seeking formal and informal mental healthcare

The sociodemographic factors associated with participants’ intentions to seek formal or informal sources of mental healthcare are displayed in Table 6. Females were more likely to seek mental health care, including from informal sources. Further, having a university education was associated with greater likelihood of seeking informal mental healthcare. Participants who identified more with their ethnic identity were more likely to seek any form of help.

**Table 6:** Likelihood of seeking informal, formal and no mental healthcare stratified by sociodemographic characteristics

	**Informal Healthcare**			**Formal Healthcare**			**No Healthcare**		
	**ß**	**95% CI**	***p*-value**	**ß**	**95% CI**	***p*-value**	**ß**	**95% CI**	***p***-value
**SLsl**	-0.031	-0.484- 0.340	0.730	-0.025	-0.529- 0.398	0.782	-0.016	-0.688- 0.580	0.866
**Age**	0.137	-0.005- 0.111	0.074	0.079	-0.031- 0.100	0.304	0.039	-0.067- 0.112	0.618
**Female**	0.160	0.110- 0.676	**0.007**	0.047	-0.190- 0.447	0.427	-0.180	-1.097- -0.226	**0.003**
**University Education**	0.130	0.059- 0.923	**0.026**	0.085	-0.125- 0.847	0.145	-0.059	-1.000- 0.330	0.322
**Single, Unmarried**	-0.067	-0.611- 0.192	0.305	-0.065	-0.677- 0.226	0.326	0.063	-0.324- 0.911	0.350
**Living at Home, with Parents**	0.051	-0.264- 0.511	0.532	-0.074	-0.636- 0.237	0.368	0.002	-0.588- 0.606	0.977
**Unemployed**	0.051	-0.708- 0.965	0.763	0.048	-0.807- 1.076	0.779	0.041	-1.134- 1.441	0.815
**Employed**	0.075	-0.635- 1.004	0.658	-0.084	-1.154- 0.691	0.621	0.022	-1.182- 1.341	0.901
**Student**	-0.083	-0.566- 0.137	0.231	-0.030	-0.483- 0.309	0.666	0.081	-0.227- 0.856	0.254
**Father University Education**	0.094	-0.070- 0.516	0.135	-0.058	-0.484- 0.175	0.357	-0.079	-0.732- 0.170	0.221
**Mother University Education**	-0.057	-0.432- 0.161	0.368	0.059	-0.176- 0.492	0.352	0.015	-0.403- 0.510	0.818
**MEIM-R**	0.149	0.039- 0.421	**0.019**	0.181	0.099- 0.529	**0.004**	-0.154	-0.651- -0.063	**0.018**
**Number of Years in Australia**	-0.112	-0.044- 0.013	0.288	-0.131	-0.052- 0.012	0.215	0.040	-0.035- 0.052	0.709
**Language Spoken at Home**	0.022	-0.120- 0.176	0.710	-0.001	-0.169- 0.164	0.980	0.048	-0.136- 0.319	0.429
**Association with Sri Lankan Groups in the Community**	-0.013	-0.0321- 0.258	0.830	0.015	-0.286- 0.366	0.807	0.004	-0.432- 0.459	0.953

*N* = 306, *MEIM-R* Multi-Ethnic Identity Measure-Revised, *SLsl* Sri Lankan Young Adults born in Sri Lanka, Numbers in bold indicate significant results. The formal and informal mental healthcare belong to the GHSQ. Refer to methods section to see comparators of variables. Adjusted *r* squared value: informal subscale = 0.084, formal subscale = 0.083

### Sociodemographic characteristics and seeking professional psychological help

The sociodemographic characteristics associated with attitudes to seeking professional psychological help is shown in Table 7.

**Table 7:** Association between attitudes towards seeking professional psychological help and sociodemographic characteristics

	**Openness**			**Value and Need**		
	**ß**	**95% CI**	***p*-value**	**ß**	**95% CI**	***p*-value**
**SLsl**	-0.119	-0.324- 0.066	0.193	0.057	-0.151- 0.295	0.525
**Age**	0.058	-0.017- 0.038	0.452	0.024	-0.027- 0.037	0.754
**Female**	0.200	0.094- 0.362	**0.001**	0.199	0.110- 0.417	**0.001**
**University Education**	-0.042	-0.278- 0.130	0.477	-0.019	-0.274- 0.195	0.740
**Single, Unmarried**	0.047	-0.121- 0.258	0.477	0.036	-0.158- 0.277	0.590
**Living at Home, with Parents**	-0.104	-0.300- 0.067	0.213	-0.056	-0.283- 0.137	0.495
**Unemployed**	0.100	-0.278- 0.513	0.559	-0.047	-0.517- 0.389	0.782
**Employed**	0.065	-0.313- 0.462	0.706	0.023	-0.414- 0.474	0.894
**Student**	-0.108	-0.297- 0.035	0.122	-0.048	-0.258- 0.123	0.484
**Father University Education**	0.040	-0.095- 0.182	0.534	0.144	0.026- 0.343	**0.023**
**Mother University Education**	0.097	-0.032- 0.248	0.129	-0.016	-0.181- 0.140	0.804
**MEIM-R**	0.144	0.013- 0.194	**0.025**	0.014	-0.092- 0.115	0.825
**Number of Years in Australia**	0.048	-0.010- 0.016	0.654	0.305	0.007- 0.038	**0.004**
**Language Spoken at Home**	0.011	-0.064- 0.076	0.861	0.001	-0.080- 0.081	0.992
**Association with Sri Lankan Groups in the Community**	-0.081	-0.228- 0.046	0.191	-0.037	-0.206- 0.108	0.541

*N* = 306, *MEIM-R* Multi-Ethnic Identity Measure-Revised, *SLsl* Sri Lankan Young Adults born in Sri Lanka, Numbers in bold indicate significant results. The openness and value subscales belong to the ATSPPH-SF. Refer to methods section to see comparators of variables. Adjusted *r* squared value: Openness scale = 0.057, value scale = 0.080

Females were significantly more open to seeking professional psychological help. Further, the more a participant positively identified with their ethnic identity, the more open they were to seeking professional psychological help.

Being female and longer duration of residence in Australia positively influenced how participants saw value and need in seeking professional psychological help. Interestingly participants with university educated fathers saw more value in seeking professional psychological help.

## Discussion

There are three main findings from this study. First, there are stigmatizing attitudes towards mental illness among Sri Lankan young adults living in Australia (SLYAs). Second, there was a difference in stigma toward MHPs and help-seeking between SLsl and SLaus. Finally, the results indicate that culture, gender and family relationships influence these attitudes and help-seeking behaviours among SLYAs.

### Strengths and limitations

A major strength of this study was the participant recruitment strategy, which was tailored to young people and mainly based online via social media. This enabled the recruitment of a large sample size of participants from both groups of SLsl and SLaus from across Australia.

A limitation of this study was its method of participant recruitment through Student Unions. As a result, we recruited a large sample of students compared to non-students in the SLsl group. International students particularly face unique stressors that affect their lives when adjusting to a completely new culture and environment in Australia [35]. For example, financial strain, accommodation satisfaction, mastering a new language, educational system and culture that is vastly different to their own [35]. These multitude of stressors may have an impact on how international students perceive mental illness and help-seeking which will be different to other students or non-students in the study sample who do not experience similar unique stressors.

### Stigmatizing attitudes and impact on help-seeking

Our findings show that SLYAs do not find help-seeking for MHPs as desirable. This could be associated with pervasive stigmatizing attitudes about mental illness and related services. When our data were compared according to country of birth, these pervasive stigmatizing attitudes were higher among SLsl in comparison to SLaus. This indicates that it is possible these attitudes are shaped in the host country. From our findings, SLsl identified more with attitudes that devalue and discriminate persons living with mental illness such as those suggested in the PSQ. This includes viewing persons living with mental illness as unintelligent, untrustworthy, irresponsible to take care of children, unemployable or viewing mental illness as a personal failure, having a lesser opinion of a person with mental illness and hesitancy to date a person with a mental illness. Our results align with a qualitative Australian study by Antoniades [6, 36] which examined Sri Lankan-Australians living with depression, finding that participants feared discrimination in employment and had negative perceptions of their intelligence if they were diagnosed with depression. It is evident that stigmatizing attitudes are underpinned by significant shame, perceptions of personal failure and potential loss of social standing within this community.

SLsl, relative to SLaus, saw significantly less value and need in seeking professional psychological help (Table 4). It is possible that this is due to the pervasive stigmatizing attitudes towards MHPs in this group. Aligning with our findings, literature on South Asian migrants have explored the link between high rates of mental illness stigma and lower help-seeking [13]. Some studies highlight that positive attitudes towards mental illness can result in a positive influence on care seeking while others highlight that positive attitudes towards help-seeking, can result in more value and willingness to seek help for MHPs [13, 37].

### Culture

Our data indicated that SLaus tended to be more open to exploring their cultural identity (Table 1) and also had significantly lower stigma toward MHPs (Table 2) and help-seeking (Table 4). Here, participants’ increased tendency to associate with their ethnicity may be due to cultural distance. It is easier to cultivate familiar cultural practices and beliefs in an alien environment [38]. Lower stigma may be due to this group of SLaus spending a majority of their lifetime in Australia where the Australian federal government dedicates a large sum yearly for investment into mental healthcare to increase access to diverse mental healthcare and progressively de-stigmatize MHPs [39, 40]. This is further supported where participants that spent more time in Australia showed lower mental illness stigma and lesser stigma towards help-seeking (Tables 5 and 7). It is possible that exposure to Australian culture and adaptation to some cultural norms may have played an important part in how SLaus showed lesser stigma toward MHPs and help-seeking.

Our data also indicate an association between ethnic identity and likelihood of seeking mental healthcare and openness to seeking professional psychological help (Tables 6 and 7). The underpinning reasons behind this association need to be further explored.

### Gender

An unexpected finding from this study was the significant impact of gender on both mental illness stigma and help-seeking. Being female was associated with lower perceived stigma towards mental illness (Table 5), lesser stigma towards help-seeking (Table 7) and higher likelihood of seeking formal and informal mental healthcare (Table 6).

Previous research by Boysen and Logan [41] explores the relationship between masculinity and mental illness stigma. The study contends that there are widely held stereotypes that men are aggressive and unemotional thereby they may perceive mental illness as a weakness or a personal failure and have more stigma toward MHPs and be less open to help-seeking. In contrast women are stereotyped as sensitive and emotionally expressive which may result in more positive perceptions of MHPs. In our data these stereotypes existed regardless of country of birth. It is possible that males may experience more pressure to adhere to their cultural values but also to conform to Australian stereotypes of masculinity [42] and this double pressure of their Sri Lankan heritage and the Anglo-Australian societal norms may affect how they perceive mental illness and help-seeking.

### Family relationships

Interestingly, we also discovered an association between participants’ fathers’ level of education and the likelihood of seeking professional psychological help, which to our knowledge, has not been explored in other studies (Table 7). The more a father was highly educated, the more the participant saw value in seeking professional psychological help. This finding reflects the stereotypical gender roles dictated by a predominantly patriarchal South Asian society [43]. Males who are often considered as the heads of households may exert a strong influence on the behaviours of the family and its responsibilities [43]. A male having a university education could possibly mean more knowledge on MHPs and the help-seeking process. This knowledge then perhaps influences how help-seeking is perceived by other members of the household. However, it is important to note that in the present study, this influence of paternal education status crossed the geographical divide and pervades in both SLsl and SLaus. The influence of fathers with less education on MHPs and help-seeking also needs to be explored.

### Implications and conclusions

The collective findings reinforce the relationship between culture, stigma and help-seeking as previously conceptualised by the literature on migrant mental health. It further highlights that mental health prevention, promotion and treatment strategies need to focus on country of birth, gender and how values in the family influence attitudes. In a public health sense, a multifaceted community-based approach should be used to gradually destigmatize mental illness and help-seeking and increase mental health service use. This could include mental health literacy programs targeted to SLYAs and their families, mental health promotion approaches involving people from the Sri Lankan community, especially those with lived experience, language services and cross-cultural communication and increasing access to publicly funded mental healthcare.

In a clinical setting, healthcare providers need to be made aware of the stigma within the community which may influence a patient’s decision to discuss mental health problems or help-seeking with a clinician. Service providers need to be assisted to avoid stereotyping, misdiagnosing or providing culturally inappropriate care, so they are able to build a relationship of trust with SLYAs.

## Data Availability

The datasets used and/or analysed during the current study are available from the corresponding author on reasonable request.

## Abbreviations

ATSPPH-SF: Attitudes towards seeking professional psychological help-short form
CALD: Culturally and linguistically diverse
GHSQ: General help seeking questionnaire
MEIM-R: Multi ethnic identity measure-revised
MHP: Mental health problem
PSQ: Perceived stigma questionnaire
SLA: Sri Lankan-Australian
SLaus: Sri Lankan young adults born in Australia
SLsl: Sri Lankan Young adults born in Sri Lanka
SLYA: Sri Lankan young adult
UN: United nations
WHO: World health organization
